# Exosomal MicroRNAs and Organotropism in Breast Cancer Metastasis

**DOI:** 10.3390/cancers12071827

**Published:** 2020-07-07

**Authors:** Grace L. Wong, Sara Abu Jalboush, Hui-Wen Lo

**Affiliations:** 1 Department of Cancer Biology, Wake Forest University School of Medicine, Winston-Salem, NC 27101, USA; glwong@wakehealth.edu (G.L.W.); sabujal@wakehealth.edu (S.A.J.); 2 Wake Forest Comprehensive Cancer Center, Wake Forest University School of Medicine, Winston-Salem, NC 27157, USA

**Keywords:** breast cancer, metastasis, exosomes, microRNAs, organotropism

## Abstract

Breast cancer is the most frequent malignancy for women in which one in eight women will be diagnosed with the disease in their lifetime. Despite advances made in treating primary breast cancer, there is still no effective treatment for metastatic breast cancer. Consequently, metastatic breast cancer is responsible for 90% of breast cancer-related deaths while only accounting for approximately one third of all breast cancer cases. To help develop effective treatments for metastatic breast cancer, it is important to gain a deeper understanding of the mechanisms by which breast cancer metastasizes, particularly, those underlying organotropism towards brain, bone, and lungs. In this review, we will primarily focus on the roles that circulating exosomal microRNAs (miRNAs) play in organotropism of breast cancer metastasis. Exosomes are extracellular vesicles that play critical roles in intercellular communication. MicroRNAs can be encapsulated in exosomes; cargo-loaded exosomes can be secreted by tumor cells into the tumor microenvironment to facilitate tumor–stroma interactions or released to circulation to prime distant organs for subsequent metastasis. Here, we will summarize our current knowledge on the biogenesis of exosomes and miRNAs, mechanisms of cargo sorting into exosomes, the exosomal miRNAs implicated in breast cancer metastasis, and therapeutic exosomal miRNAs.

## 1. Introduction

Breast cancer is the most frequently diagnosed cancer and the second leading cause of cancer-related deaths in women [1]. Metastatic malignancies account for 20–30% of breast cancer cases with a five-year survival rate of 22%; those metastatic cases are responsible for 90% of breast cancer deaths [2,3]. Breast cancer can be histologically classified into pre-invasive (ductal carcinoma in situ) and invasive (ductal carcinoma). The intrinsic, molecular subtypes of invasive breast carcinomas are clinically relevant classifications, which include triple-negative breast cancer (TNBC), human epidermal growth factor receptor 2 (HER2)-enriched (nonluminal), luminal B-like HER2-positive, luminal B-like HER2-negative, and luminal A breast cancer [2,4]. TNBC is classified due to the lack of estrogen receptor (ER), progesterone receptor (PR), and HER2 receptor. HER2-enriched breast cancer is defined by the increased expression of the HER2 receptor and is associated with aggressive proliferation, intermediate to poor prognosis, and limited therapies. Luminal A and luminal B are the least aggressive breast cancer subtypes that tend to be ER and PR-positive, HER2 expression variable, and can be distinguished based on their proliferation rates (Ki67 index) [2]. TNBC and HER2-enriched breast cancers have the highest propensity to metastasize to distant organs, such as brain, bone, lung, liver, and the abdominal cavity [4,5]. Current metastatic breast cancer treatments are limited to surgical resection, chemotherapy, radiotherapy (whole brain or organ, depending on the metastatic location), stereotactic radiotherapy, and systemic therapy [6,7,8]. All therapeutic approaches are dependent on the clinical diagnoses, gene expression profile, and location(s) of metastases. Despite advances in breast cancer therapeutics, clinical management of the metastatic disease remains challenging and this is, in part, attributed to our limited knowledge of the mechanisms that drive breast cancer metastasis and those underlying organ site-specific metastasis.

Exosomes are extracellular vesicles of endosomal origin that range in size from ~30–100 nm in diameter. Originally termed “exosomes” when membrane fragments were found to be isolated from biological fluids, exosomes are now widely studied for their important roles in communication and waste removal. Exosomes are composed of a lipid bilayer that encapsulates a myriad of bioactive molecules including small noncoding RNAs, lipids, nucleic acids, and proteins. When exosomes are released from multivesicular bodies (MVBs), they are known to travel throughout body fluids (blood, saliva, urine, etc.) and release the contents of their compartments upon fusion with the plasma membrane of target cells [9,10,11,12,13]. Exosomes play important roles in intra- and intercellular communication, such as removal of harmful or damaged cell components or stimulation of immune cells [14]. Due to their critical roles in cell–cell communication, tumor-derived exosomes have been implicated as major players in the crosstalk between cancer cells and new, potential microenvironments, termed “pre-metastatic niches” [10,13,15,16]. 

MicroRNAs (miRNAs) are small, ~21–23 nucleotide (nt), endogenous noncoding RNAs that have been shown to play regulatory roles in gene expression in many physiological processes [17,18]. In 1993, the first identified miRNA, transcribed from the *lin-4* gene, was found to produce a functional transcript that did not encode a protein, but instead exhibited antisense, suppressive activity of the protein-coding gene *lin-14* [19]. Five years later, the RNA interference (RNAi) mechanism was discovered with small-interfering RNAs (siRNAs) as the effectors of this widely utilized mechanism, which was awarded the Nobel Prize in Physiology or Medicine eight years later [20]. MiRNAs are synthesized as double-stranded precursors in the nucleus, cleaved (multiple times, sequentially), and translocated to the cytoplasm. Mature miRNAs are loaded onto Argonaute proteins (AGOs) to produce RNA-induced silencing complexes (RISC) that target messenger RNAs (mRNAs) via partial complementary base pairing in the 3′-untranslated region (3′-UTR) or 5′-UTR [18,19,21,22,23]. 

Recently, miRNAs have been shown to play regulatory roles in numerous biological pathways involved in development, proliferation, differentiation, and cell death [14,17,24]. Transported miRNAs, such as those utilizing exosomes as vehicles, have been found to be upregulated in cancer, especially regulating pathways involved in cancer proliferation, growth, and metastasis. Since the discovery of the first miRNA in 1993, thousands of human miRNAs have been identified to play important roles in many cancer types including breast cancer [10,13,14,15,16,25,26]. This review will summarize recent discoveries in the fields of exosome biogenesis, miRNA biogenesis, cargo sorting into exosomes, and the exosomal miRNAs that have been reported to regulate breast cancer organ site-specific metastasis. Additionally, this review will discuss potential novel therapeutic applications of these exosomal miRNAs for breast cancer patients. 

## 2. Exosomes, MicroRNAs, and Packaging

### 2.1. Exosome Biogenesis

In 1983, two research labs each published the discovery of extracellular vesicles (EVs), later termed “exosomes”, when investigating the transferrin receptor in the maturation of reticulocytes [27,28]. A dissertation focused on the role of the transferrin receptor in the maturation of reticulocytes identified the pathway in which the transferrin receptor was recycled between the plasma membrane and the endocytic compartments [9]. Through this investigation, they discovered that the transportation of these transferrin receptors involved a smaller class of vesicles, now known as intraluminal vesicles (ILVs), which are formed through the invagination of the early endosome membrane. These ILVs were discovered to form from larger, mature endosomes, referred to as MVBs, that can fuse with either the lysosome for degradation and recycling or with the plasma membrane to release their contents to the extracellular space [29]. Those vesicles that encapsulate the distributed cargo to the extracellular space are exosomes.

Formation of MVBs requires the endosomal sorting complex required for transport (ESCRT), which is a complex of four proteins (ESCRT-0–III) that all facilitate MVB formation, budding, and cargo distribution [30,31,32]. Initiation of the ESCRT pathway involves the ubiquitination of ESCRT-0 that promotes binding to cargo-containing endosomes. ESCRT-I then binds to the N terminus end of ESCRT-0, while ESCRT-II binds the other end to form the trimeric ESCRT complex. This trimeric ESCRT complex initiates membrane budding and packaging. Binding of ESCRT-II initiates the recruitment of the final ESCRT (ESCRT-III) to the endosome where the ESCRT-III subunits, Vps20 and Snf7, facilitate vesicular budding in an ATP-dependent manner that directs membrane scission from the cytoplasmic side [14,31,33,34]. 

Additional players identified in cargo packaging and exosome biogenesis include the ALG-2-interacting protein X (ALIX) and the associated syndecans and syntenin, tumor susceptibility gene 101 (TSG101), charged multivesicular body protein 4 (CHMP4, also termed Snf7), CHMP6 (also termed Vps20), CHMP3 (also termed Vps24), LIP5 (also termed Vtla1), and Vps4 [33,35,36]. Vacuolar protein sorting (Vps) factors, conserved throughout eukaryotes, mostly function on the cytosolic side of endosomal membranes and assist in sorting cargo into vesicles as subunits of many of the ESCRT complexes [36]. Furthermore, ALIX has been shown to interact with both ESCRT-I and ESCRT-III in cargo sorting and facilitate the entire process of vesicular budding. These proteins, among others, are still being identified as key players in membrane budding and scission processes, such as endosome sorting, cytokinesis, enveloped virus budding, and growth factor receptor endocytosis [36,37]; however, these players and mechanisms go beyond the scope of this review. 

ILV-containing MVBs can direct distribution of the ILVs by targeting them to the lysosome for degradation or by fusing with the plasma membrane to release the cargo-containing exosomes to the interstitial space [30,32]. At the plasma membrane, Rab GTPases, specifically RAB27A and RAB27B, mediate exocytosis of the ILV-containing MVBs. Furthermore, inhibition of RAB27A in a mouse metastatic breast carcinoma resulted in decreased primary tumor growth and delayed metastases in vivo [38]. Interestingly, inhibition of RAB27A in a mouse nonmetastatic breast carcinoma resulted in no significant difference in tumor growth or time to metastases of the primary tumor as compared to the control group. Moreover, RAB27A knockout cell line resulted in significantly decreased exosome secretion in TNBC cells [39]. These findings suggested an important role for RAB27A in mediating tumor-secreted exosomes in breast cancer metastasis. 

While directed transport and docking of the MVBs is facilitated by Rab GTPases, both soluble N-ethylmaleimide sensitive factor attachment protein receptor complexes (SNAREs) and Sec1/Munc18-like (SM) proteins act cooperatively to mediate membrane fusion [40]. SNAREs are divided into two categories: v-SNAREs, which mediate vesicular transport, and t-SNAREs, located on target membranes to facilitate the MVB membrane fusion with the target cell. Interactions between the v-SNAREs and t-SNAREs generate an inward force vector, which mediates membrane fusion that assists in overcoming the topological barriers associated with these interactions [14,40]. Following fusion of the MVB to the target membrane, ILVs are released into the extracellular space as cargo-containing exosomes.

### 2.2. MicroRNA Biogenesis

In 1993, Victor Ambros’ lab discovered that the *lin-4* gene in the model organism *Caenorhabditis elegans* did not encode a protein, but instead a functional RNA transcript that silenced the LIN-14 protein via antisense RNA-RNA interactions [19,22]. At the time, the noncoding portion of any organism’s genome was considered “junk” and deemed useless. Attention to these molecules referred to as “noncoding RNAs” made way for the discovery of RNA interference (RNAi) under the direction of Craig Mello, who’s siRNA-based mechanism was awarded the Nobel Prize in Physiology or Medicine in 2006 [20,41]. As the field of noncoding RNAs grew, the Human Genome Project was completed in 2003 to mark the first, fully sequenced human genome, thwarting the myth of “junk” regions of the genome as the project revealed that only approximately 1% of the human genome encodes proteins [42]. 

Similar to other small noncoding RNAs, classical miRNA activity targets mRNAs for cleavage or sequestration leading to mRNA degradation. Since the identification of the first miRNA, thousands of miRNAs have been discovered in the human body that have been found to regulate over 30% of human protein-coding genes [43,44]. Unlike siRNAs, miRNAs are encoded in the human genome and have been found to be derived from introns or long noncoding RNAs (lncRNAs) [45]. Currently, the main miRNA database, named miRBase, reports 1917 miRNA precursors (referred to as pre-miRNAs) with over 2000 mature sequences in the human genome [46,47]. MiRNAs can be categorized into families based on the resemblance of their seed sequences, which are the 2–8 bases that are the primary effectors in binding complementary target sequences [48]. 

A critical aspect of mature miRNA effector function is the Argonaute protein (AGO) that associates with the miRNA and directs to sequence-specific targets. Mature miRNAs are loaded onto these AGOs and create the miRNA-induced silencing complex (miRISC) that carries out RNAi functions [46,49,50]. Eukaryotic AGOs have highly conserved RNAi-based functions that are carried out by four domains (N, PAZ, MID, and PIWI) produced by one polypeptide chain [50,51,52]. The human genome contains four AGO proteins (AGO1-4), which have been shown to confer some degree of specificity, although most mature miRNAs can interact with all four AGOs. AGO2 is the predominant AGO in humans and confers the “slicer” activity that comprises the miRISC’s ability to target and cleave mRNA transcripts [49]. Interestingly, AGO2 expression has been shown to be regulated by the epidermal growth factor receptor (EGFR) and mitogen-activated protein kinase (MAPK) pathways in breast cancer cell lines [53]. More specifically, epidermal growth factor (EGF) increased AGO2 expression and stability at the protein level in metastatic TNBC cells. These findings suggest the importance of AGOs, as well as miRNAs, in breast cancer and that metastatic breast cancer cells may be able to upregulate both oncogenic miRNAs and their effector AGOs. 

MiRNAs are transcribed primarily by RNA Polymerase II as precursor hairpin (pre-miRNA) structures in the nucleus. The pre-miRNAs are processed by the Microprocessor complex, which consists of the RNAse III endonuclease Drosha, and the DiGeorge syndrome critical region 8 (DGCR8) [54,55,56]. The canonical mechanism of pre-miRNA export utilizes Exportin 5, which recognizes the 2-nt overhang at the 3′-end (characteristic of many small noncoding RNAs) and facilitates the export into the cytoplasm [57]. Following export to the cytoplasm, Dicer, the cytoplasmic RNAse III enzyme, processes the pre-miRNAs into species-specific double-stranded RNA molecules that can be loaded onto active AGOs [43,46,58,59]. RNAses Drosha and Dicer represent critical factors for both miRNA biogenesis and embryonic development, as deletions in these foundational genes are lethal in mouse embryos [46,60,61,62]. Moreover, Dicer has been shown to be essential for normal mouse development because of its role in blood vessel formation and maintenance [63]. Additionally, alternative processing by Dicer can result in mature miRNA variants (referred to as “isomirs”) that add complexity and specificity to miRNA functions. Recently, these isomirs were evaluated similar to gene expression profiles and revealed that isomer signatures could be utilized to discriminate between 32 TCGA-based cancer types [64]. Overall, the complex biogenesis and effector functions of miRNAs reveal fundamental roles in a tumorigenic environment and warrant further investigation into the mechanisms contributing to breast cancer metastasis.

### 2.3. Mechanisms of Cargo Sorting into Exosomes

The molecular mechanisms of miRNA sorting and packaging into exosomes are still not well understood. Two major categories of mechanisms that have been described include nonselective and selective miRNA sorting [39]. Nonselective miRNA sorting was identified in larger exosomes when comparing a breast cancer cell line to normal breast epithelium; however, the method used did not include further purification into subpopulations, which have been suggested to be highly heterogeneous [65]. The first described selective miRNA sorting requires heterogeneous nuclear ribonucleoprotein A2B1 (hnRNPA2B1) where the presence or absence of sumoylation determines miRNA-loading [66]. The next described mechanism of selective miRNA sorting utilizes a cell-free method to identify miRNA species selection and packaging into exosomes [67]. In this in vitro method, selective sorting of miR-223 requires the RNA-binding Y-box protein I (YBX1). The third described mechanism involves the synaptotagmin-binding cytoplasmic RNA-interacting protein (SYNCRIP or hnRNP-1 or NSAP1) and a short, conserved sequence (hEXO motif) that is implicated in miRNA exosome loading [68]. 

Another mechanism of cargo sorting revealed that the transfer of exosomal domains into particular endosome regions requires neutral sphingomyelinase-2 (nSmase2), and not the ESCRT complexes as previously predicted [26,69]. This ESCRT-independent mechanism involves the accumulation of sphingolipids that form ceramides that can induce domain budding within the endosome. The last suggested mechanism for cargo sorting into exosomes utilizes the 3′ end of the miRNA, which is most often adenylated or uridylated [70]. These post-transcriptional modifications, identified through comprehensive bioinformatics, may contribute to exosomal packaging mediated by miRISCs. Furthermore, AGO2 was implicated as a mediator of this mechanism; however, later research revealed that AGO2 is not packaged into exosomes, but may be secreted in a nonexosomal mechanism [39,71]. Nonetheless, cargo sorting mechanisms into exosomes, whether highly selective or not, need further investigation as the mechanisms still remain unclear. 

Interestingly, a study focused on miRNA sorting revealed that two biochemically distinct exosome populations are released from TNBC cells [39]. Exosomal miR-122, among a few others, was identified in the classical exosome population and followed the selective mechanism of miRNA sorting. The same study revealed that the highly selected miRNAs carried in TNBC-secreted exosomes do not contain AGO2 or Dicer [39]. These findings suggest that the miRNAs are transported as double-stranded molecules and are loaded onto AGOs, most likely AGO2, at the premetastatic niche. It has also been suggested that miRNA sorting could represent an important step of regulation for tumor cells if they could selectively secrete exosomes loaded with oncogenic miRNAs (termed “oncomiRs”) and dispose of miRNAs with tumor suppressive activity. 

## 3. Roles of Exosomal MicroRNAs in Breast Cancer Metastasis

Exosomes have been implicated in cell–cell communication via mediation of surface-membrane trafficking and horizontal transfer of bioactive molecules between tumor cells and cellular components in the surrounding tumor microenvironment [9,29,32,72,73]. More specifically, exosomes are involved in biological events leading to tumor metastasis. These events include tumor cell proliferation, motility, recruitment and activation of many oncogenic cell types (or converted cancer-promoting cell types), transfer of oncogenic bioactive molecules, increase in angiogenesis, invasion, and immunosuppression to evade cell death [11,14,73,74,75,76,77].

Simultaneously, numerous labs have now demonstrated the importance of miRNAs in mediating cellular responses via regulation of gene expression [21,24,46,78,79]. One of the earliest large-scale studies utilized high volume bioinformatics data analysis to not only identify human oncomiRs and tumor-suppressive miRNAs, but also to categorize them based on a number of characteristics including function, conservation, genome pattern localization, targets, transcriptional regulators, and even predicted target genes [80]. Results from this study revealed that oncomiR genes were favorably located in amplified regions, in contrast to tumor-suppressive miRNA genes that tended to be localized to deleted regions. Similar studies have allowed researchers to identify hundreds of novel miRNAs in many cancer types [81,82,83,84]. Further, it is well established that in order for primary tumor cells to metastasize to distant organs, tumor cells must interact with the surrounding microenvironment. The microenvironment, which contributes to tumorigenic success or failure, can include stromal cells, immune cells, endothelial cells, fibroblasts, stem cells or progenitor cells, and extracellular matrix. MiRNAs play major roles in mediating important processes that determine metastatic potential, such as proliferation, migration, invasion, epithelial-to-mesenchymal (EMT) transition, mesenchymal-to-epithelial (MET) transition, matrix-metalloproteinase (MMP) activity, angiogenesis, immune cell evasion, and cell death [85,86,87,88]. However, the mechanisms by which primary tumor cells modulate and “prime” sites for metastases warrant further examination. The literature discussed in the next sections has reported how tumor cells manipulate premetastatic sites by the use of exosome-mediated transfer of miRNAs. Investigating these exosomal miRNAs provides valuable mechanistic insights into breast cancer metastasis and could present researchers with potential therapeutic targets.

**Figure 1:**
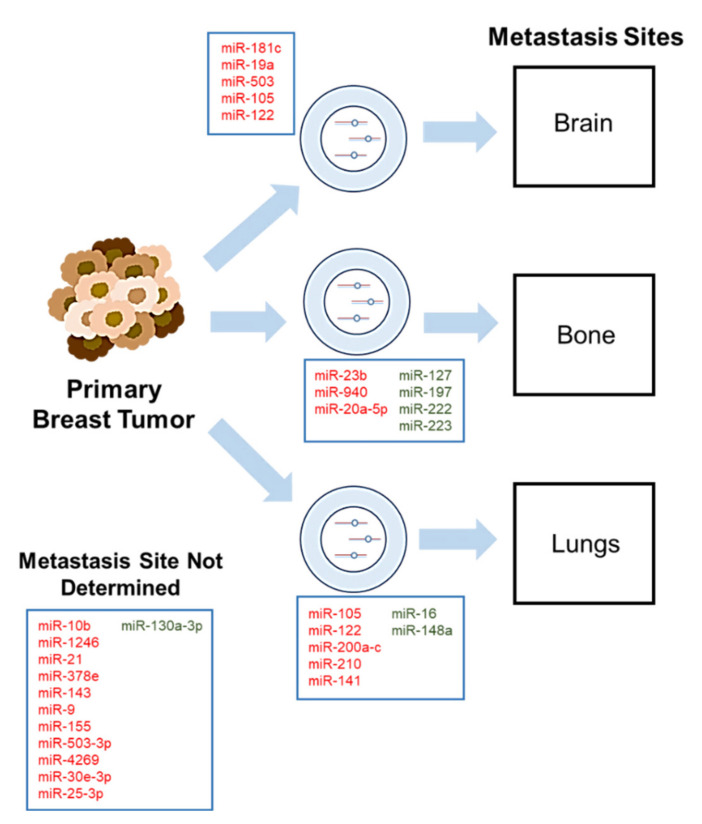
Exosomal microRNAs mediate breast cancer metastasis to preferential premetastatic niches: brain, lungs, and bone. Primary breast tumor cells communicate and prime premetastatic niches via exosome-mediated transfer of miRNAs. Manipulated microenvironments by breast cancer cells have been established in both in vivo mouse models and observed in clinical practice. Many exosomal miRNAs have been implicated in organ-specific breast cancer metastasis, though not all have established metastasis sites. MiRNAs in red demonstrate oncogenic roles whereas miRNAs in green demonstrate tumor-suppressive roles.

Exosomal miRNAs have been shown to act as both tumor suppressors and oncogenes in breast cancer. An important aspect that contributes to these metastases includes the tumor microenvironment, which was first proposed with the “seed and soil” hypothesis in 1889 [89,90]. This hypothesis implicates the tumor microenvironment as a major player and states that communication between specific tumor types that retain metastatic potential (“seed”) and a specific organ microenvironment or niche (“soil”) is hardly random. This hypothesis, later termed “organotropism”, has been supported in breast cancer since it was suggested that breast cancer metastasizes to specific organs, namely the brain, bone, and lungs [89,90,91,92,93]. This section of the review will be discussed based on each individual breast cancer metastasis niche: brain, bone, and lungs (Figure 1 and Table 1) and will conclude with exosomal miRNAs identified in breast cancer that currently do not have determined metastatic niches, but provide evidence of their involvement in breast cancer metastasis.

### 3.1. Exosomal MicroRNAs in Breast Cancer Brain Metastasis

More aggressive breast cancer subtypes, such as HER2-enriched and TNBC, have high metastatic potential and predictably metastasize to the brain [3]. A clinical study with 191 breast cancer brain metastasis (BCBM) patients revealed that the overall survival for the entire cohort was less than 5 years, and that BCBM patients with the HER2-enriched tumors and TNBC had the shortest survival times of 4.3 and 3.2 years, respectively [94]. A SEER (Surveillance, Epidemiology, and End Results database) population-based study showed that breast cancer patients with brain metastases had the highest hazard ratio (impact on survival with synchronous metastases) of all the patients [95]. Brain metastases occur in 10–30% of metastatic breast cancer patients with BCBM representing 15% of all brain metastasis cases [96,97]. Treatment options for brain metastases have improved beyond surgery, such as radiotherapy, chemotherapy and stereotactic radiosurgery; however, more effective therapies for these patients are urgently needed. To help meet this need, it is important to gain new insights into the mechanisms of brain metastasis development and progression [97]. 

In 2013, a study indicated that brain metastatic-derived exosomes could be taken up by nonmetastatic breast cancer cells, paving the way for exosomal roles in brain metastasis [98]. Most recently, a tumor-specific transcription factor known as truncated glioma-associated oncogene homolog 1 (tGLI1), discovered in 2009, was shown to promote BCBM in TNBC by upregulating stemness-associated genes and activating astrocytes in the brain [99,100]. Though mechanistic details are under investigation, preliminary results revealed that exosomal microRNAs may provide the intercellular communication needed to prime astrocytes prior to BCBM establishment. Exosomal miRNAs have been shown to regulate tumor angiogenesis, a critical aspect of tumor metastasis [88]. For example, exosomal miR-105, one of the earliest discovered exosomal miRNAs in breast cancer, could promote tumor cell migration by targeting ZO-1, a critical endothelial cell tight junction protein [101]. Utilizing brain metastatic TNBC cells and xenograft tumor models, exosome-secreted miR-105 was demonstrated to disrupt endothelial barriers and promote metastasis in poorly metastatic tumors in miR-105-overexpressing cells. 

In addition to angiogenesis, metastasis to the brain involves penetration of the blood–brain barrier (BBB). The BBB protects and regulates the central nervous system homeostasis by forming a molecular barrier around the brain [102,103]. The BBB primarily consists of endothelial cells, pericytes, astrocytes, and microglia referred to as the regulatory neurovascular unit. Breast cancer cells have been shown to breach the BBB via modulation by vascular endothelial growth factor (VEGF) to increase brain metastatic potential in TNBC cells [104]. Moreover, extracellular vesicles have been shown to penetrate the BBB via transcytosis and provide a vehicle that may be utilized by primary breast tumors to metastasize to the brain [105]. Exosomal miR-181c, enriched and secreted by brain metastatic TNBC cells, promoted the destruction of the BBB by targeting PDPK1 and promoted brain metastasis in vivo [106]. PDPK1 downregulation resulted in abnormal actin localization and destruction of the BBB.

In 2015, the first report of astrocyte-secreted exosomes carrying miRNAs targeting the critical tumor suppressor PTEN increased metastatic tumor cell proliferation and brain metastasis [13]. Utilizing brain metastatic TNBC cells with both intracarotid and intracardiac mouse models, PTEN expression was shown to be targeted by astrocyte-derived exosomal miR-19a, evident when inhibition of miR-19a activity or inhibition of astrocyte exosome secretion resulted in both rescue in PTEN expression as well as suppression of brain metastasis in vivo [13]. This groundbreaking discovery provided a new perspective on BCBM mechanisms [107,108]. 

In a similarly compelling and thorough study, exosomal miR-122 was shown to enhance the Warburg Effect in premetastatic niches, specifically the brain and lungs, in TNBC cells [109]. Primary tumor-derived exosomes carrying miR-122 downregulated PKM, a glycolytic pyruvate kinase, to decrease glucose uptake by nontumorigenic cells in the premetastatic niches to promote metastasis by increasing nutrient availability. Additionally, exosomal miR-503 has been shown to activate the M1 to M2 transformation of microglia in the brain [108]. Loss of X-inactive specific transcript (XIST), a lncRNA associated with BCBM, enhanced exosomal miR-503 secretion that resulted in upregulated immunosuppressive cytokine secretion and inhibited T-cell proliferation, promoting BCBM. While these exosomal miRNAs have been identified as key players of BCBM, many of the pathological mechanisms that facilitate metastasis to the brain remain unclear.

### 3.2. Exosomal MicroRNAs in Breast Cancer Bone Metastasis

A SEER population-based study performed from 2010 to 2015 revealed that 5% of all carcinoma patients reported presentation of bone metastases at the time of diagnosis [95]. Of the over 2 million recorded patients, the overall 5-year survival significantly decreased with the presence of bone metastases including breast cancer patients. Additionally, breast cancer patients that presented with more than one distant organ metastasis (such as brain and bone or lungs and bone, etc.) had a 5-year survival rate drop to 32% from 86% compared to those without metastases. Thus, identifying mechanisms that promote bone metastases from primary breast tumors could provide important therapeutic targets for breast cancer patients. 

While mechanisms by which primary breast tumors metastasize to the bone are still not well understood, it has been noted that bone metastases can recur after ten years of remission, suggesting that metastasized breast cancer cells may lie dormant in the bone for significant periods of time [110,111]. Multiple miRNAs (miR-127, -197, -222, and -223), through transfer by exosomes and gap junctions, contributed to a dormant phenotype in breast cancer bone metastases by targeting CXCL12, a stromal-cell derived chemokine ligand [110]. Similarly, exosomal miR-23b could promote dormancy of bone marrow-metastatic breast cancer cells by targeting MARCKS [112]. This study revealed that co-culture of TNBC cells with bone-marrow mesenchymal stem cells suppressed proliferation of the TNBC cells, as well as, decreased invasion and the presence of stem cell markers. Furthermore, growth of breast cancer xenografts was significantly decreased following treatment with exosomes containing miR-23b as compared to their control cohort [112]. 

Exosomal miRNAs have also been shown to induce noncharacteristic phenotypes in certain cell types. Exosomal miR-940 could induce osteogenic differentiation associated with an osteoblastic phenotype in TNBC cells [113]. Additionally, miR-940-overexpressing TNBC cell lines implanted on the calvarial bones or injected into the tibia of nude mice exhibited extensive mineralization and osteoblastic lesions in vivo. Exosomal miR-20a-5p could promote proliferation and migration in TNBC cells and facilitate osteoclastogenesis in mouse bone marrow-derived macrophages by suppressing SRCIN1, a Src protein kinase involved in regulation of cell migration [114]. Taken together, these findings suggest an important role for exosomal miRNAs in regulating the bone metastatic environment.

### 3.3. Exosomal MicroRNAs in Breast Cancer Lung Metastasis

Primary breast tumors, depending on their intrinsic classification, also have the propensity to metastasize to the lungs. Interestingly, TNBC has been reported to maintain the highest propensity to metastasize to distant organs, and more specifically has an increased probability of spreading to the lungs first [5]. It is estimated that 60% of metastatic breast cancer patients will be diagnosed with lung or bone metastases, while almost 70% of metastatic breast cancer patients that will succumb to the disease would have a prior lung metastasis diagnosis [115,116]. 

Exosomal miR-200 and miR-141 have been shown to regulate the mesenchymal-to-epithelial transition (MET), a critical process in metastatic colonization at a distant site, by targeting SEC23A, ZEB2, and CDH1 [91,117]. This study revealed that exosome-secreted miR-200 promoted colonization of circulating murine breast cancer cells and could transform nonmetastatic cells to promote metastasis in vivo [91]. Exosomal miR-141, which can be categorized within the miR-200 family based on similarity of the seed sequence, exhibited similar functions to miR-200, though at less significant impacts. Exosomal miR-210 was implicated in mediating enhanced angiogenesis from TNBC cells, although exact targets remain unidentified [118]. Exosomal miR-200c, a miR-200 variant, was associated with increased tumor progression and subsequent lung metastasis over time [119]. 

In contrast, exosomal miR-16 and exosomal miR-148a were observed to exhibit tumor-suppressive functions in breast cancer [120]. In this study focused on the role of focal adhesion kinases (FAKs) in cancer-associated fibroblasts (CAFs), deletion of FAKs significantly inhibited CAF-mediated exosome secretion. Furthermore, significant increase in exosomal miR-16 and miR-148a expression in FAK-null CAFs resulted in a significant decrease in migration and proliferation when treated with miRNA inhibitors, allowing the authors to conclude that both miRNAs played an integral role in tumor suppression. 

Additionally, both exosomal miR-105 and miR-122, described earlier in the brain metastasis section, have also been shown to promote metastasis to the lungs by targeting vascular endothelial barriers and suppressing glucose uptake by nontumorigenic cells, respectively [101,109]. Taken together, these reports indicate the important roles that exosomal miRNAs play in breast cancer lung metastasis.

### 3.4. Exosomal MicroRNAs in Breast Cancer Migration, Invasion and Stemness

Metastasis is a multi-step process involving EMT, migration, invasion, intravasation, extravasation, MET, and colonization. While breast cancer stem cells tend to be more metastatic, a number of exosomal miRNAs have also been shown to regulate some of the premetastatic steps and stem cell renewal. For example, exosomal miR-10b, secreted from TNBC cells, induced invasion in nonmalignant mammary epithelial cells by down-regulating HOXD10, associated with the homeobox protein family [26]. Similarly, TNBC-derived exosomal miR-1246 was shown to promote invasion and proliferation by targeting CCNG2, an important cyclin-dependent protein kinase [121]. Moreover, exosomal miR-25-3p increased the migration phenotype in TNBC cells in a hypoxic environment [122]. Inhibition of miR-25-3p suppressed tumor growth, while inhibition of miR-25-3p and treatment of HIF-1α repressed tumor growth as compared to treatment with HIF-1α alone in vivo. Exosomal miR-1910-3p could promote proliferation, migration, and autophagy, while inhibiting apoptosis in TNBC and ER- and PR-positive breast cancer cells [123]. This extensive study revealed that miR-1910-3p, contained in TNBC and ER- and PR-positive breast cancer cells, inhibited MTMR3, subsequently activating the NF-κB signaling pathway. Interestingly, tumor-associated lymphatic vessels contributed to increased invasion and migration by secreting exosomes containing miR-503-3p, -4269, and 30e-3p in TNBC cells [124]. All three exosomal miRNAs were identified as targets of ELK3, an ETS domain-containing protein with both transcriptional activation and repression capabilities downstream of the ERK1/2 and Ras signaling pathways. Inhibition of ELK3 in vitro resulted in decreased invasion and migration and suppressed TNBC cell-derived tumor growth in vivo. 

Furthermore, exosome-secreted miR-21, -378e, and -143 were reported to promote breast cancer cell stemness by increasing mammosphere forming capabilities, stem cell expression markers, EMT markers, and anchorage-independence [75]. In contrast, exosomal miR-130a-3p suppressed breast cancer stem cell proliferation, invasion and migration by targeting RAB5B, a RAB family GTPase [125]. Normal breast tissue exhibited significantly higher levels of exosomal miR-130a-3p as compared to breast cancer tissue samples; however, no in vivo assays were performed with miR-130a-3p or RAB5B. 

Exosomal miRNAs have been shown to act directly on target genes to induce or suppress specific events as well as play roles in the modulation of critical pathway regulators, such as tumor suppressor genes. For example, tumor suppressors phosphatase and tensin homolog (PTEN) and dual specificity phosphatase 14 (DUSP14) could be targeted by exosome-secreted miR-9 and miR-155, respectively (Table 1) [126]. These findings suggest that exosomal miRNAs could play key roles in regulating tumorigenic transformation, though further investigation into these roles is necessary.

## 4. Exosomal MicroRNAs as Cancer Therapeutics 

Given the important roles that miRNAs exhibit in tumor progression, therapeutic miRNAs are making moves toward FDA-approval [20,42,127]. Currently, seven miRNA drugs are in clinical trials with three of those drugs reaching phase II [78]. These interventional clinical trials address viable therapeutic treatments for human diseases with no other effective treatments. Despite new biotech companies devoted to developing miRNA therapeutics, such as Miragen, MiRNA Therapeutics, and Regulus Therapeutics, delivery and drug resistance provide the largest hurdles in the field. First, delivery of these miRNAs (or miRNA mimics) as potent effector molecules can confer severe side effects without effective target specificity [78,127]. Similar to classical systemic drugs, miRNAs can be encapsulated in liposomes, nanoparticles, and micelles; however, they have a limited ability to penetrate the blood–brain barrier [78]. MiRNAs can provide promising clinical applications, however, no miRNA drug has yet to make it to a phase III clinical trial as many of the phase II trials were terminated early due to severe adverse events [78,127,128].

Most primary breast tumors respond to initial treatments, but develop resistance months after initial response [129,130]. To overcome this resistance, exosomal miRNAs and exosomes transporting other molecular cargo have been explored as second-line treatments for refractory breast cancer in light of their essential role in tumor drug resistance (Table 2) [131]. For example, exosome-mediated miR-567 was tested against HER2-enriched, trastuzumab-resistant breast cancer cell lines as a method to reverse autophagy-dependent chemoresistance [132]. Resistance to trastuzumab was overcome by treatment with miR-567, which directly targets ATG5, a critical protein for autophagy execution, in vivo. Interestingly, shikonin, a naphthoquinone extracted from traditional Chinese medicine, was found to suppress breast cancer cell proliferation by inhibiting exosome secretion [133]. The exosomes targeted by shikonin contained miR-128, which has been associated with decreased levels of Bax, inhibiting the miRNAs ability to suppress apoptosis. These findings suggest a role for exosomes as vehicles for treatments as well as potential targets for breast cancer therapeutics. 

Exosomal miR-423-5p was demonstrated to confer resistance to cisplatin in TNBC cells [134]. Exosome-secreted miR-222 rendered Adriamycin-sensitive breast cancer cells resistant to Adriamycin; conversely, miR-222 inhibitors resulted in loss of Adriamycin resistance [135]. Moreover, exosomal miR-222, along with miR-221, also conferred Tamoxifen resistance of Tamoxifen-sensitive breast cancer cells through the downregulating p27 and ERα [136]. 

Exosomal miRNAs have also been found to be downregulated in breast cancer cells as compared to their normal, healthy counterparts. Exosomal miR-134 is significantly downregulated in breast tumor tissues; overexpression of exosomal miR-134 significantly suppressed TNBC cell proliferation and increased cisplatin-induced apoptosis, which is in part attributed to a decrease in STAT5B and subsequent suppression of Hsp90 and Bcl-2 [137]. Additionally, endothelial-derived exosomal miR-503 transferred to TNBC cells could suppress tumor cell proliferation and invasion by targeting CCND2 and CCND3 [138]. This study also revealed that treatment with neoadjuvant chemotherapy led to increased miR-503 levels in the endothelial-derived exosomes, but significantly downregulated in the endothelial cells. Exosomal miR-770 overexpression enhanced doxorubicin sensitivity in TNBC cell lines via induction of apoptosis [139]. Moreover, miR-770 overexpression suppressed migration and invasion of TNBC cells by targeting STMN1, a stathmin family phosphoprotein involved in intracellular signaling, in vitro. This study’s tumor xenograft model utilizing TNBC cells overexpressing miR-770 in combination with treatment of doxorubicin revealed significantly decreased tumor volume and metastasis compared to the control cohort. These studies provide the rationale to further investigate the importance of exosomal miRNAs in breast cancer metastasis and drug resistance, and develop exosomal miRNA-based cancer therapeutics. 

## 5. Conclusions

Since the discovery of the first miRNA and the completion of the Human Genome Project, the notion that noncoding RNAs are “junk” has been proven incorrect. Although exosomal miRNAs have been found to contribute to biological processes that underlie most human cancers, absences and inconsistencies in the field are still major obstacles in a clinical setting. First, scientific approaches to isolating extracellular vesicle subgroups are variable across laboratories. Additionally, extracellular vesicle subgroups have not been defined as certain laboratories identify multiple classes of exosomes, while others group exosomes as distinct from shedding vesicles, apoptotic bodies, and larger MVBs [14,39]. Second, the mechanisms of miRNA sorting and exosome biogenesis are also variable and not well understood. Third, exosome populations have been noted to be heterogeneous, making distinct, standardized expression or surface markers difficult to confirm. Finally, exosome secretion, exosome levels, miRNA levels, and general tumor-specific responses all vary depending on the patient. Further investigation is required to elucidate the mechanisms by which tumor cells or the surrounding tumor microenvironment utilize or regulate exosomal miRNAs in clinical practice. Exosomes are 30–100 nm extracellular vesicles that maintain critical roles in intercellular communication. Exosomes carry a variety of bioactive molecules including RNA, DNA, proteins, and lipids. MiRNAs are small noncoding RNA molecules that play regulatory roles in almost every biological process in the human body. MiRNAs transferred from exosomes have been shown to play important roles in breast cancer metastasis. More specifically, these exosomal miRNAs can mediate metastasis to established premetastatic niches in breast cancer that include the brain, bone, and lungs. Because of their ability to interact with their surrounding environment, exosomal miRNAs can promote or regulate the tumor microenvironment. These exosomal miRNA-mediated responses warrant further investigation in breast cancer metastasis as findings may provide a myriad of opportunities for targeted breast cancer therapies in the future.

## Figures and Tables

**Table 1:** Exosomal miRNAs mediate organotropism in breast cancer metastasis.

Exosome-Secreted miRNAs	Activity	Validated Targets	In Vitro Model(s)	Additional Model(s)	Metastatic Site(s)	Reference
miR-9	Enriched in TNBC, suppresses PTEN (tumor suppressor)	PTEN*	Human BC cell lines: MDA-MB-231, MCF-7, MCF-10A	N/A	ND	[126]
miR-10b	Promotes invasion	HOXD10*, KLF4	Human BC cell lines: MDA-MB-231, MCF-7, MCF-10A, HMLE	N/A	ND	[26]
miR-16	Tumor suppressive activity (in FAK null-CAFs)	N/A	Human BC cell lines: MDA-MB-231, MCF-7, WI-38	In vivo (transgenic mice)	Lungs	[120]
miR-19a	Increases tumor growth and brain metastasis formation	PTEN*	Human BC cell lines: MDA-MB-231, HCC1954, BT474, MDA-MB-435; mouse cell lines: 4T1, B16BL6	In vivo (intracarotid, intracranial models)	Brain	[13]
miR-20a-5p	Promotes proliferation and migration of BC cells; facilitates osteoclastogenesis	SRCIN1*	Human BC cell lines: MDA-MB-231, MCF-7, MCF-10A; BMMs	Clinical samples	Bone	[114]
miR-21	Increased in CAF-derived exosomes; promotes stemness, EMT, and anchorage-independence	N/A	Human BC cell lines: BT549, MDA-MB-231, T47D	Clinical samples (TCGA)	ND	[75]
miR-23b	Promotes dormancy phenotype	MARCKS*	Human BC and BM-MSCs: MDA-MB-231, R14, R36, R37, 4F0218	In vivo (MFP injections), clinical samples	Bone	[112]
miR-25-3p	Promotes proliferation and migration of BC cells in a HIF-1-dependent manner	N/A	Human BC cell line: MDA-MB-231; mouse cell lines: E0771, RAW264.7 (macrophage cell line)	In vivo (MFP injections)	ND	[122]
miR-30e-3p	Regulated by ELK3; promote migration and invasion	N/A	Human BC cell lines: MDA-MB-231, Hs-578T, BT-20, MCF-7, MCF-10A; LECs, HUVEC	In vivo (orthotopic inoculation), databases	ND	[124]
miR-105	Promotes metastasis by targeting vascular endothelial barriers	ZO-1	Human BC cell lines: MDA-MB-231, MCF-10A; HMVECs	In vivo (MFP injection, tumor xenograft model)	Lungs, Brain	[101]
miR-122	Suppress glucose uptake in non-tumorigenic cells to promote metastasis	PKM*	Human BC cell lines: MDA-MB-231, MCF-10A	In vivo (tail vein and intracardiac injections)	Lungs, Brain	[109]
miR-127	Tumor suppressive activity: bone marrow mets transmit miRNAs to BC cells via gap junctions and exosomes	CXCL12*	Human BC cell lines: MDA-MB-231, T47D	N/A	Bone	[110]
miR-130a-3p	Tumor suppressive activity: decreased levels associated with lymph node metastasis, O/E inhibited cell proliferation, migration, invasion	RAB5B*	Human BC cell lines: MDA-MB-231, MCF-7, MCF-10A, BCSCs	N/A	ND	[125]
miR-141	Regulates MET (suppresses EMT), which promotes lung metastasis and colonization	SEC23A*, ZEB2, CDH1	Human BC cell lines: MCF-10A, MDA-MB-231; mouse cell lines: 4T1, 4T07, 67NR	In vivo (tail vein injection)	Lungs	[91]
miR-141	miR-141 is regulated by FOXP3-KAT2B axis, promotes tumor metastasis	N/A	Human BC cell lines: MCF-7, T47D, BT474, MDA-MB-468	In vivo (transgenic mice), patient samples	Lungs	[119]
miR-143	Increased in CAF-derived exosomes; promotes stemness, EMT, and anchorage-independence	N/A	Human BC cell lines: BT549, MDA-MB-231, T47D	Clinical samples (TCGA)	ND	[75]
miR-148a	Tumor suppressive activity (in FAK null-CAFs)	N/A	Human BC cell lines: MDA-MB-231, MCF-7, WI-38	In vivo (transgenic mice)	Lungs	[120]
miR-155	Enriched in TNBC, suppresses DUSP14 (tumor suppressor)	DUSP14*	Human BC cell lines: MDA-MB-231, MCF-7, MCF-10A	N/A	ND	[126]
miR-181c	Promotes breakdown of BBB through abnormal localization of actin; promotes brain metastasis	PDPK1*	Human BC cell lines: MDA-MB-231, BMD1a, BMD2a, BMD2b	In vivo (intracardiac injection and tail vein)	Brain	[106]
miR-197	Tumor suppressive activity: bone marrow mets transmit miRNAs to BC cells via gap junctions and exosomes	CXCL12*	Human BC cell lines: MDA-MB-231, T47D	N/A	Bone	[110]
miR-200 (a, b, c)	Regulates MET (suppresses EMT), which promotes lung metastasis and colonization	SEC23A*, ZEB2, CDH1	Human BC cell lines: MCF-10A, MDA-MB-231; mouse cell lines: 4T1, 4T07, 67NR	In vivo (tail vein injection)	Lungs	[91,117]
miR-200c	miR-200c is regulated by FOXP3-KAT2B axis, promotes tumor metastasis	N/A	Human BC cell lines: MCF-7, T47D, BT474, MDA-MB-468	In vivo (transgenic mice), patient samples	Lungs	[119]
miR-210	Enhances angiogenesis	N/A	Human BC cell lines: MDA-MB-231, MCF-10A; mouse cell line: 4T1	In vivo (subcutaneous and MFP injections)	Lungs	[118]
miR-222	Tumor suppressive activity: bone marrow mets transmit miRNAs to BC cells via gap junctions and exosomes	CXCL12*	Human BC cell lines: MDA-MB-231, T47D	N/A	Bone	[110]
miR-223	Tumor suppressive activity: bone marrow mets transmit miRs to BC cells via gap junctions and exosomes	CXCL12*	Human BC cell lines: MDA-MB-231, T47D	N/A	Bone	[110]
miR-378e	Increased in CAF-derived exosomes; promotes stemness, EMT, and anchorage-independence	N/A	Human BC cell lines: BT549, MDA-MB-231, T47D	Clinical samples (TCGA)	ND	[75]
miR-503	Promotes M1 to M2 polarization of microglia (results in up-regulation of suppressive cytokines that suppress T-cell proliferation)	STAT3	Human BC cell lines: MCF-7, SKBR3, 231 BRM and SKBRM (brain metastasis cell lines derived from MDA-MB-231 and SKBR3, respectively)	Databases (GEO), in vivo (intracardiac injection)	Brain	[108]
miR-503-3p	Regulated by ELK3; promote migration and invasion	N/A	Human BC cell lines: MDA-MB-231, Hs-578T, BT-20, MCF-7, MCF-10A; LECs, HUVEC	In vivo (orthotopic inoculation), databases	ND	[124]
miR-940	Facilitate osteogenic differentiation of host mesenchymal cells (osteoblastic phenotype)	ARHGAP1*, FAM134A* (validated in osteosarcoma line)	Human BC cell line: MDA-MB-231	In vivo (implant onto calvarial bones/skull or injection into tibia)	Bone	[113]
miR-1246	Promotes proliferation, invasion	CCNG2*	Human BC cell lines: MDA-MB-231, MCF-7, MCF-10A, HMLE	N/A	ND	[121]
miR-1910-3p	Promotes proliferation, migration, metastasis, and autophagy	MTMR3*	Human BC cell lines: MDA-MB-231, MCF-7, MCF-10A; 293T (human embryonic kidney cells)	In vivo (subcutaneous xenograft models), clinical samples	ND	[123]
miR-4269	Regulated by ELK3; promote migration and invasion	N/A	Human BC cell lines: MDA-MB-231, Hs-578T, BT-20, MCF-7, MCF-10A; LECs, HUVEC	In vivo (orthotopic inoculation), databases	ND	[124]

* Indicates validation with luciferase assay and 3′ UTR of the reported target gene. ND: not determined; N/A: not applicable; BC: breast cancer; BBB: blood-brain barrier; BMM: bone marrow macrophages; HMVECs: human microvascular endothelial cells; MET: mesenchymal-to-epithelial transition; EMT: epithelial-to-mesenchymal transition. CAFs: cancer-associated fibroblasts; BM-MSCs: bone marrow mesenchymal stem cells; O/E: overexpression; BCSCs: breast cancer stem cells; MFP: mammary fat pad; TNBC: triple-negative breast cancer; LECs: lymphatic endothelial cells; HUVECs: human umbilical vein endothelial cells.

**Table 2:** Exosomal miRNAs implicated in drug resistance or treatment efficacy.

Exosome-Secreted miRNAs	Drug	Expression Level	Donor Cell	Recipient Cell	Activity	Target Gene(s)	Reference
miR-134	17-AAG, PU-H71	Decreased	MCF-7/Resistant	MCF-7/Sensitive	Reduced cell proliferation, invasion, migration and increased cisplatin-induced apoptosis	STAT5B, Hsp90, Bcl-2	[137]
miR-221/222	Tamoxifen	Increased	MCF-7/Resistant	MCF-7/Sensitive	Increased tamoxifen resistance	p27, ERalpha	[136]
miR-222	Adriamycin	Increased	MCF-7/Resistant	MCF-7/Sensitive	Gained adriamycin-resistance when transfected with miR-222 mimics, lost resistance with miR-222 inhibitors	N/A	[135]
miR-423-5p	Cisplatin (DDP)	Increased	MDA-MB-231	MCF-7, SKBR3	Increased cell proliferation, migration, and cisplatin resistance	P-gp	[134]
miR-503	Epirubicin, Paclitaxel	Decreased	HUVEC	MDA-MB-231	Suppressed tumor cell proliferation and invasion	CCND2*, CCND3	[138]
miR-567	Trastuzumab	Decreased	N/A	SKBR3/R, BT474/R	Inhibits autophagy, reverses chemoresistance	ATG5*	[132]
miR-770	Doxorubicin (DOX)	Decreased	MDA-MB-231, MDA-MB-468	MDA-MB-231, THP-1	Increased doxorubicin sensitivity and induced apoptosis; suppressed tumor cell migration and invasion	STMN1*	[139]
miR-1246	Docetaxel (DTX), Epirubicin (EPI), Gemcitabine (GEM)	ND	MDA-MB-231	HMLE	Increased cell apoptosis after treatment with DTX, EPI, and GEM	CCNG2*	[121]

* Indicates validation with luciferase assay and 3’ UTR of the reported target gene. N/A: not applicable (not determined); BC: breast cancer; EVs: extracellular vesicles; CSC: cancer stem cell.
